# Influence of Different Application Modes of a Universal Adhesive System on the Bond Strength of Bulk‐Fill Composite Resin to Enamel and Dentin in Primary Teeth

**DOI:** 10.1002/cre2.947

**Published:** 2024-08-28

**Authors:** Ali Nozari, Maryam Pakniyat Jahromi, Farnaz Haji Abbas Oghli, Zahra Jowkar, Seyed Ahmadreza Hamidi

**Affiliations:** ^1^ Department of Pediatric Dentistry, School of Dentistry Shiraz University of Medical Sciences Shiraz Iran; ^2^ Oral and Dental Disease Research Center, Department of Operative Dentistry, School of Dentistry Shiraz University of Medical Sciences Shiraz Iran; ^3^ Department of Operative Dentistry, School of Dentistry Shiraz University of Medical Sciences Shiraz Iran

**Keywords:** bulk‐fill composite resin, primary teeth, universal adhesive system

## Abstract

**Objectives:**

The objective of this study was to assess how the application mode of a universal adhesive system affects the microshear bond strength (μSBS) of bulk‐fill and conventional composite resins to enamel and dentin in primary teeth.

**Methods:**

A total of 80 caries‐free primary second molars were randomly assigned to eight experimental groups (*n* = 10) based on the bonding substrate (enamel or dentin), the application mode of the universal adhesive system (etch and rinse [E&R], or self‐etch [SE]), and the type of composite resin used (bulk‐fill or conventional). After bonding the composite resin to enamel or dentin, the μSBS of the bonded composite resins was measured.

**Results:**

The mean μSBS value of bulk‐fill composite resin was significantly higher than that of conventional composite resin for both enamel and dentin substrates, regardless of the application mode (*p* < 0.001). An interaction effect between the bonding substrate and the application mode of the adhesive system was observed, indicating a significant relationship (*p* < 0.001). The highest μSBS values for primary teeth enamel were achieved using the E&R mode with bulk‐fill composite resin, while for dentin specimens, the SE mode with bulk‐fill composite resin yielded the highest μSBS values. The μSBS of the E&R group was significantly higher than that of the SE group for enamel specimens (*p* < 0.001), whereas the μSBS of the SE group was significantly higher than that of the E&R group for dentin specimens (*p* < 0.001).

**Conclusion:**

Bulk‐fill composite resin demonstrated higher μSBS in comparison to conventional composite resin. The universal adhesive system exhibited superior performance in the SE mode compared to the E&R mode on primary dentin. Pre‐etching the enamel before the application of the universal adhesive enhanced the μSBS to primary teeth enamel, highlighting the importance of selectively acid etching the enamel of primary teeth.

## 1 Introduction

The utilization of composite resins for the conservative treatment of carious primary teeth located at the posterior region has gained significant popularity (Poggio et al. 2013). Despite their favorable esthetic outcomes and minimal removal of tooth structure, composite resins are susceptible to shrinkage during the curing process. This shrinkage can lead to detachment at the interfaces between the tooth, adhesive, and composite, creating opportunities for bacterial infiltration and resulting in microleakage at the site of restoration (Poggio et al. 2013; Nozari et al. 2019). To address the issues associated with polymerization shrinkage, the incremental layering technique has been proposed as a clinical approach (Santis et al. 2020). However, this technique increases the chances of void formation between composite resin layers, raises the risk of contamination, necessitates precise execution, and extends the duration of the dental procedure (Kim et al. 2015).

In recent years, there has been an emergence of bulk‐fill composite resins with the purpose of reducing stress at the adhesive interface (Lima et al. 2018). These bulk‐fill composite resins are equipped with a highly efficient initiator system and excellent translucency, allowing for a substantial depth of cure. Consequently, they can be applied in a single layer of up to 4 mm, thereby reducing the overall treatment time required for cavity filling (Lima et al. 2018). Notably, bulk‐fill composite resins have been found to exhibit reduced polymerization shrinkage compared to traditional composite resins. This reduction can be attributed to the incorporation of stress‐relieving monomers and pre‐polymerized particles in bulk‐fill composite resins (Fronza et al. 2015). Bulk‐fill composite resins can be categorized as either flowable or packable (Ilie, Bucuta, and Draenert 2013). Flowable bulk‐fill composite resins, due to their low viscosity, can be injected into the cavity and adapt well to the cavity walls. However, they tend to have lower wear resistance and often require an additional layer of conventional composite resin (Ilie, Bucuta, and Draenert 2013). In contrast, packable bulk‐fill composites obviate the requirement for an additional top layer and enable the restoration of the complete cavity in a single application (Ilie, Bucuta, and Draenert 2013).

Reducing the amount of time spent in the dentist's chair is crucial in pediatric clinical practice, as it helps decrease the chances of contamination and improves the cooperation of young patients (Oter, Deniz, and Cehreli 2018). As a result, the use of packable bulk‐fill composite resins has been suggested for the efficient and convenient restoration of significant cavities in primary teeth (Ilie et al. 2014). It is worth highlighting that findings derived from research conducted on permanent teeth cannot be directly extrapolated to primary teeth because of inherent dissimilarities in their shape and chemical makeup. Consequently, the results observed in studies involving permanent teeth may not precisely represent the behavior or response of primary teeth under similar conditions or treatments. Fewer investigations have been conducted on the effectiveness of bulk‐fill composite resins in primary teeth when compared to permanent teeth (Oter, Deniz, and Cehreli 2018; Ehlers et al. 2019). A prior investigation revealed that the shear bond strength of bulk‐fill composite resins to primary enamel and dentin was either similar to or greater than that of nanohybrid composite resin (Ilie et al. 2014). Furthermore, the application of bulk‐fill composite resin, specifically Tetris N‑Ceram Bulk‑Fill composite from Ivoclar Vivadent, demonstrated a significant improvement in the fracture resistance of primary molars following pulpotomy, when compared to a traditional composite resin, as evidenced by a previous study (Ghajari et al. 2020).

The latest generation of adhesive systems is commonly known as universal adhesives, also referred to as “multimode” or “multipurpose” adhesives. These universal adhesives contain all the necessary bonding components in a single bottle and can be used in either the etch‐and‐rinse (E&R) or self‐etch (SE) mode (de Goes, Shinohara, and Freitas 2014). The effectiveness of universal adhesives in primary teeth has been examined in a limited number of studies. In a previous investigation, it was discovered that the application of a universal adhesive system (Scotchbond Universal Adhesive) using the SE approach resulted in lower bond strength values to primary dentin compared to the E&R method (Lenzi, Soares, and de Oliveira Rocha 2017). However, an alternative study demonstrated that the bond strength of two universal adhesive systems (Scotchbond Universal and All‐Bond Universal) to primary tooth dentin was increased by prior acid etching (Kim et al. 2017). In contrast, a study by Nicoloso et al. found no significant difference in bond strength between the SE and E&R approaches (Nicoloso 2016). As a result, there is ongoing debate regarding the impact of application mode on the bond strength of universal adhesives to primary teeth (Lenzi et al. 2013; Nicoloso 2016; Thanaratikul, Santiwong, and Harnirattisai 2016; Kim et al. 2017).

To the best of the authors' knowledge, no prior investigation has explored the impact of the application mode of universal adhesives on the bond strength of bulk‐fill composite resins in primary teeth. Hence, the objective of this in vitro study was to evaluate how the application mode of a universal adhesive system affects the microshear bond strength (μSBS) between a bulk‐fill composite resin and the enamel and dentin surfaces of primary teeth. The study aimed to test the null hypothesis that there would be no significant differences in μSBS when using different application modes of the universal adhesive system to bond the bulk‐fill composite resin to the enamel and dentin surfaces of primary teeth.

## 2 Materials and Methods

### 2.1 Tooth Preparation

Following approval from the Research and Ethics Committee of Shiraz University of Medical Sciences (Protocol #IR.SUMS.DENTAL.REC.1401.024), a total of 80 primary second molars, free from caries, cracks, or enamel defects, were obtained. These teeth had been extracted for orthodontic purposes. The parents of the patients were informed about the study's purpose and the use of the extracted teeth, and they provided informed consent by signing relevant forms. All experimental procedures were performed by a trained operator who was unaware of the study conditions. The collected teeth were cleaned using a periodontal curette and then stored in a 0.5% chloramine T solution at a temperature of 4°C. The storage duration did not exceed 1 month before the teeth were utilized for the study.

### 2.2 Specimen Preparation

The roots and crowns of all specimens were separated and the roots were discarded. Forty of the samples (crowns) were allocated for the assessment of μSBS to enamel, while the remaining 40 were specifically designated for evaluating dentin bond strength.

**Figure 1:**
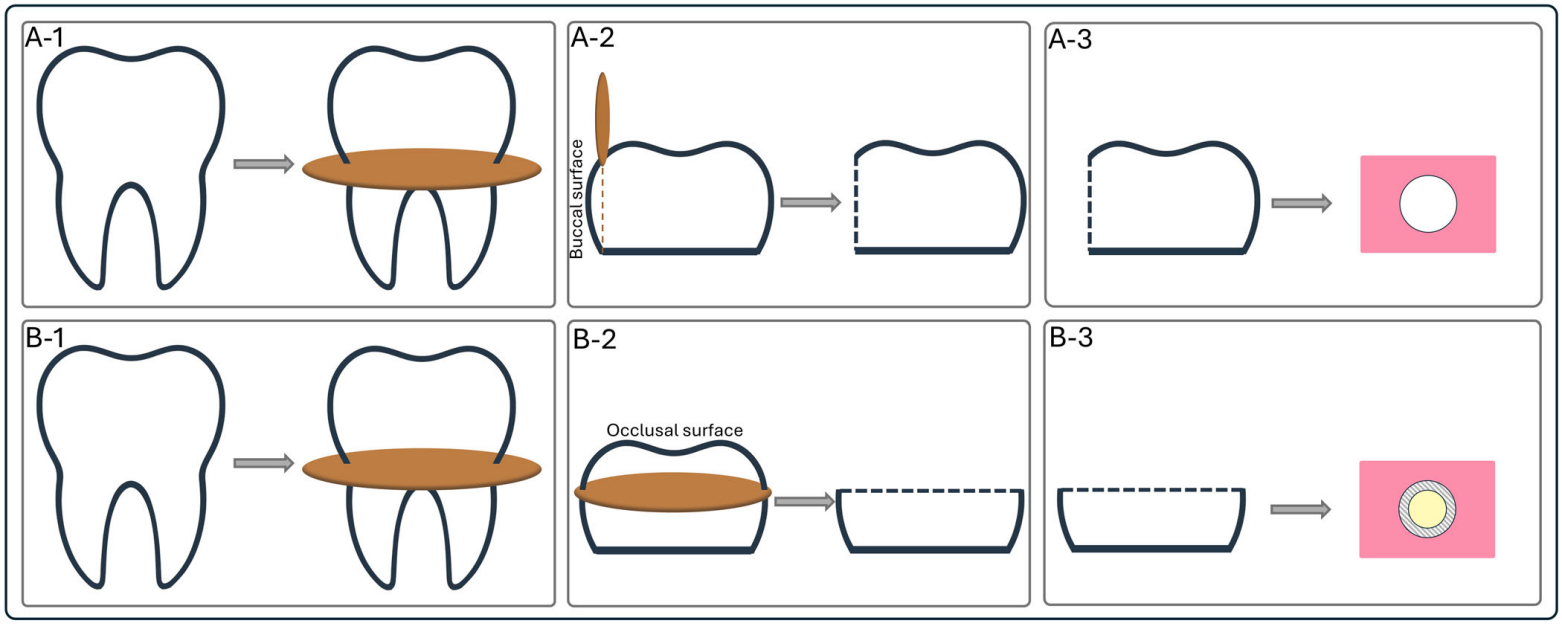
Schematic drawing depicting the process of preparing enamel and dentin specimens: (A‐1) Specimens undergoing separation of roots and crowns, with the roots being subsequently discarded, (A‐2) preparation of enamel specimens involved creating flat enamel surfaces with a depth of 0.5 mm at the midbuccal regions of tooth crowns, (A‐3) prepared specimens mounted in acrylic resin blocks, positioned with the prepared buccal surfaces facing upward and parallel to the base of the resin blocks; (B‐1) Separating and discarding the roots from the specimens, (B‐2) preparation of dentin specimens involved creating flat midcoronal dentin surfaces by removing the occlusal enamel and superficial dentin, (B‐3) mounting of prepared dentin specimens in acrylic resin blocks, with the dentin surfaces positioned parallel to the base of the resin blocks.

Figure 1 displays a schematic diagram illustrating the procedure involved in preparing enamel and dentin specimens. To prepare the enamel specimens, flat enamel surfaces with a depth of 0.5 mm were created at the midbuccal regions of the tooth crowns using a low‐speed cutting machine (Mecatome T201 A, Presi, Grenoble, France). Adequate water cooling was ensured during the preparation process. Subsequently, the prepared specimens were mounted in acrylic resin blocks, with the buccal surfaces facing upward and parallel to the base of the resin blocks. To achieve standardized flat enamel surfaces, the prepared areas were slightly wet‐ground using 320‐grit silicon carbide papers. The presence of dentin on the enamel surfaces was confirmed using a stereoscopic microscope (Carl Zeiss, Oberkochen, Germany).

For the dentin specimens, flat midcoronal dentin surfaces were prepared. The occlusal enamel and superficial dentin were removed from the samples using a low‐speed cutting machine (Mecatome T201 A, Presi, Grenoble, France) with water‐cooling. This process was carried out to create dentin specimens. The prepared dentin specimens were then mounted in acrylic resin blocks, with the dentin surfaces aligned parallel to the base of the resin blocks. To generate a consistent smear layer on the prepared surfaces of the specimens, 320‐grit silicon carbide papers were used for 1 min. Following that, the specimens were rinsed and dried using an air–water syringe.

### 2.3 Experimental Groups

**Figure 2:**
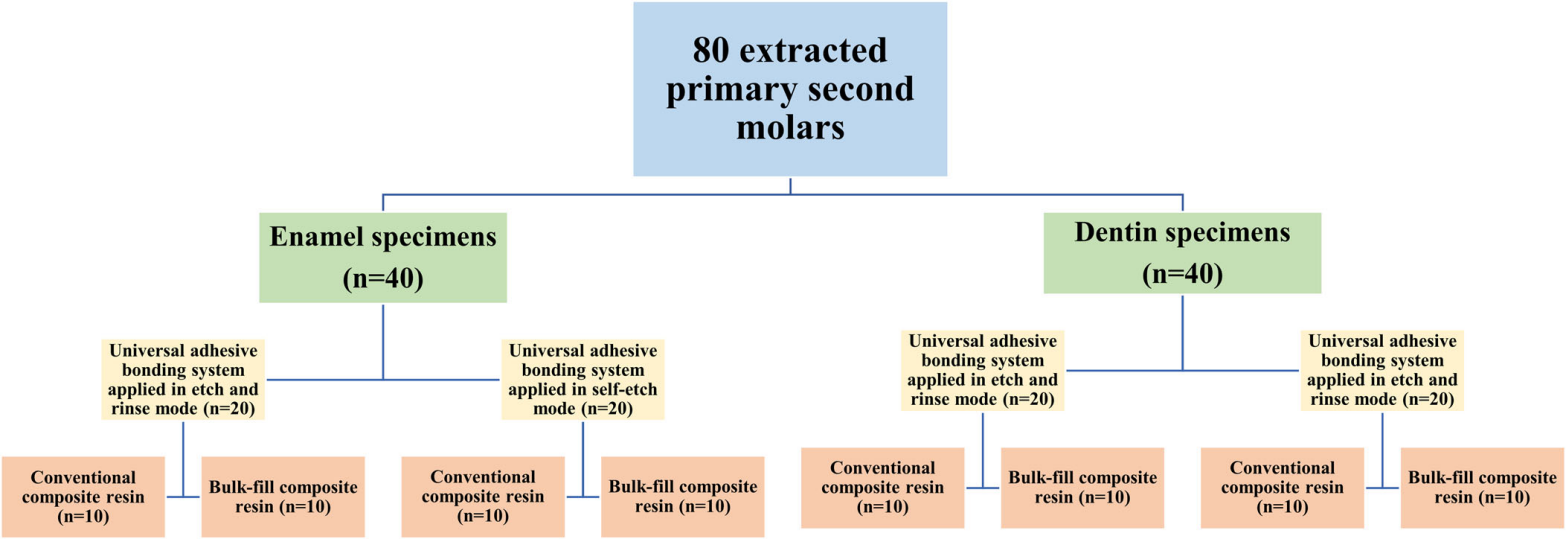
The study groups and their corresponding study protocols in a visual format.

The prepared specimens were randomly allocated into eight experimental groups, with each group consisting of 10 samples. The grouping was determined by three factors: the bonding substrate (enamel and dentin), the mode of application of the universal adhesive bonding (E&R or SE), and the type of composite resin (conventional or bulk‐fill). Figure 2 visually represents the study groups and their respective study protocols. Materials used in this study are presented in Table 1.

**Table 1:** Materials specifications.

Material	Type	Manufacturer (Batch No.)	Composition	Application procedure
Gluma Bond Universal	Universal adhesive bonding system	Heraeus Kulzer GmbH, Hanau, Germany (M010057)	MDP phosphate monomer, 4‐META, dimethacrylate resins, acetone, fillers, initiators, silane	Etch‐and‐rinse mode: 1) For enamel specimens, apply Phosphoric acid etching gel (37%) to enamel and leave undisturbed for 20 s; for dentin specimens, apply Phosphoric acid etching gel (37%) to dentin and leave undisturbed for 15 s 2) Rinse the prepared surface using air–water spray for 30 s and dry with absorbent paper 3) Apply the adhesive to the entire prepared surface with the applicator brush and rub for 20 s 4) Dry sufficiently by blowing mild air for more than 5 s until the adhesive resin does not move 5) Light‐cure for 10 s SE mode: 1) Apply the adhesive to the entire prepared surface with the applicator brush and rub for 20 s 2) Dry sufficiently by blowing mild air for more than 5 s until the adhesive resin does not move 3) Light‐cure for 10 s
Grandio	Conventional composite resin nano‐hybrid	Voco, Cuxhaven, Germany (2120406)	Resin matrix: Bis‐GMA, TEGDMA Filler type: Barium–boron–alumino–silicate glass (0.1–2.5 μm), Silica: 20–60 nm Filler content: 87%wt	1) Apply Grandio in layers of no more than 2 mm thickness 2) Light cure for 20 s
x‐tra fil	Bulk‐fill composite resin (microhybrid)	Voco, GmbH, Cuxhaven, Germany (2202165)	Resin matrix: Bis‐GMA, UDMA, TEGDMA Filler type: Barium–boron–alumino–silicate glass (2–3 μm) Filler content: 86%wt	1) Apply x‐tra fil in layers of no more than 4 mm thickness 2) Light cure for 20 s

Abbreviations: 4 META, 4‐methacryloxyethyl trimellitate anhydride; 10‐MDP, 10‐methacryloyloxydecyl dihydrogen phosphate; Bis‐GMA, bisphenol A glycidyal dimethacrylate; TEGDMA, triethyleneglycol dimethacrylate. UDMA, urethane dimethacrylate.

Groups 1, 3, 5, and 7 involved the application of the universal adhesive bonding system (Gluma Bond Universal; Heraeus Kulzer GmbH, Hanau, Germany) using the E&R mode as per the manufacturer's instructions. Conversely, groups 2, 4, 6, and 8 utilized the universal adhesive bonding system in the SE mode, following the manufacturer's instructions.

In terms of the composite resin selection, groups 1, 2, 5, and 6 applied a conventional composite resin (Grandio; Voco, Cuxhaven, Germany) after the application of the adhesive bonding system. In contrast, groups 3, 4, 7, and 8 utilized a bulk‐fill composite resin (x‐tra fil, Voco, GmbH, Cuxhaven, Germany).

### 2.4 Microshear Bond Strength and Failure Mode Analysis

**Figure 3:**
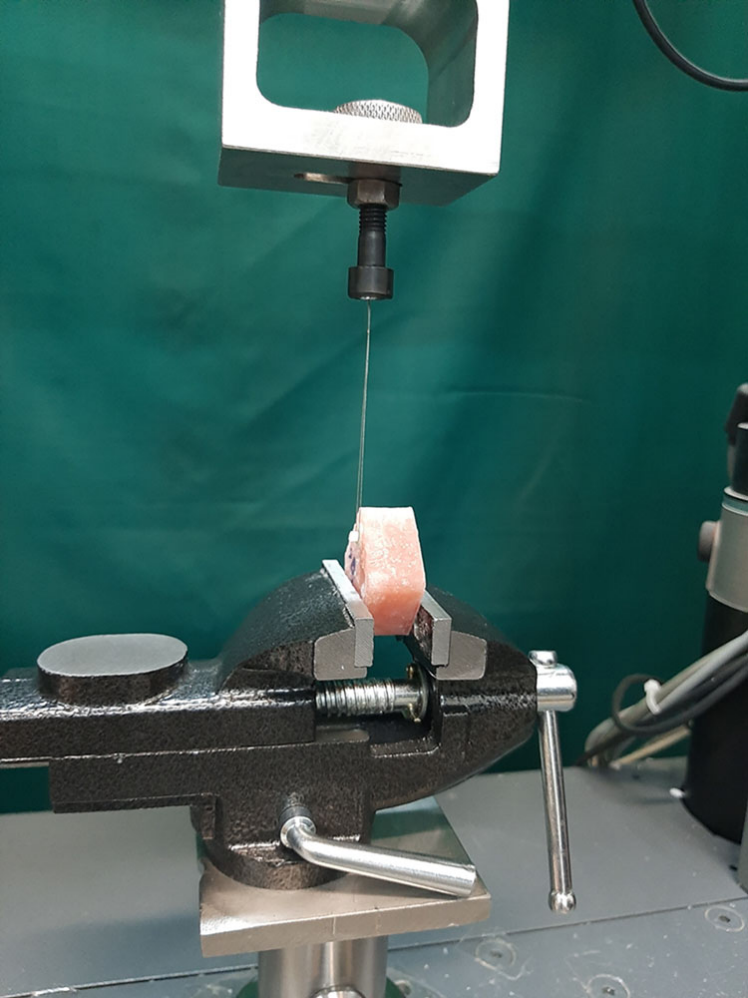
A bonded specimen with wrapped around stainless steel ligature wire to the base of a composite resin micro‐cylinder under the universal testing machine.

To demarcate the bonding area on the prepared dentin or enamel surfaces, adhesive tape with a hole was employed. A translucent microtube made of polyvinyl chloride, measuring 0.5 mm in height and 0.7 mm in internal diameter, was positioned over the punched hole of the adhesive tape. Subsequently, the adhesive bonding was light cured, and the composite resin was placed into the microtubes, followed by another round of light curing. The light curing was performed using a light curing unit (VIP Junior, Bisco, Schaumburg, IL, USA) with a power density of 600 mW/cm². The bonded specimens were then submerged in distilled water at 37°C for 24 h. The μSBS between the composite resins and the enamel or dentin was assessed using a universal testing machine (Instron, Z020, Zwick Roell, Germany) at a crosshead speed of 1 mm/min (Figure 3). The load at which each specimen failed was recorded and divided by the bonded surface area to calculate the bond strength values in megapascals (MPa).

To analyze the failure modes, the surfaces where debonding occurred were inspected using a stereomicroscope (Carl Zeiss Inc., Oberkochen, Germany) at a magnification of ×40. The failure modes were classified into three categories: (A) adhesive failure within the adhesive interface, (B) cohesive failure within the composite resin or dental substrate (enamel or dentin), and (C) mixed failure involving both adhesive and cohesive components.

### 2.5 Statistical Analysis

The normality of the data was assessed using the Shapiro–Wilk test. As the data demonstrated a normal distribution, a three‐way analysis of variance (ANOVA) model was utilized to analyze the data and assess the impacts of the three primary factors: bonding substrate (enamel or dentin), type of composite resin, and mode of application of the universal bonding. Subsequently, *T* tests were conducted to compare the μSBS values between various groups. Statistical analyses were carried out using SPSS software version 17 (SPSS Inc., Chicago, USA). A significance level of 0.05 was employed, indicating that *p* values below this threshold were considered statistically significant.

## 3 Result

**Figure 4:**
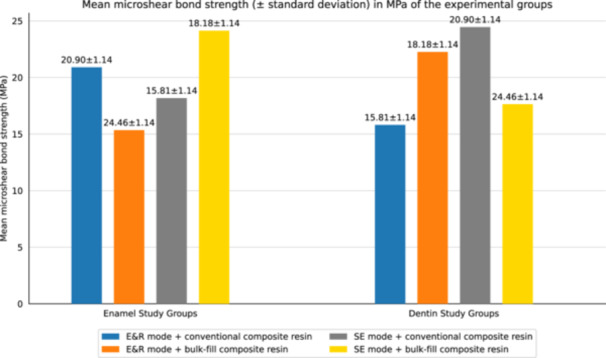
Bar chart depicting the microshear bond strength of the study groups.

Table 2 displays the mean μSBS values (with standard deviation [SD]) in megapascals. Figure 4 illustrates a bar chart that displays the μSBS of the various study groups. The results of the three‐way ANOVA indicated notable variations among the experimental groups (*p* < 0.05). The type of composite resin also displayed a significant impact (*p* < 0.001) according to the three‐way ANOVA. *T* tests revealed a significant increase in the mean μSBS value of the bulk‐fill composite resin compared to the conventional composite resin. This significant difference was observed in both enamel and dentin substrates, as well as in both modes of application (*p* < 0.001). Furthermore, the three‐way ANOVA identified a significant interaction effect between the bonding substrate and the mode of application of adhesive bonding (*p* < 0.001), as demonstrated in Table 3. Independent *t* tests were conducted for pairwise comparisons of μSBS values across different groups.

**Table 2:** Mean microshear bond strength (± standard deviation) in MPa of the enamel experimental groups.

Group number	Group description	Mean microshear bond strength (± standard deviation)
1	Enamel + E&R mode + conventional composite resin	20.90 ± 1.14
2	Enamel + SE mode + conventional composite resin	15.35 ± 1.15
3	Enamel + E&R mode + bulk‐fill composite resin	24.46 ± 1.18
4	Enamel + SE mode + bulk‐fill composite resin	17.64 ± 1.23
5	Dentin + E&R mode + conventional composite resin	15.81 ± 1.02
6	Dentin + SE mode + conventional composite resin	22.26 ± 1.53
7	Dentin + E&R mode + bulk‐fill composite resin	18.18 ± 1.38
8	Dentin + SE mode + ulk‐fill composite resin	24.14 ± 1.82

Abbreviations: E&R, etch and rinse; SE, self‐etch.

**Table 3:** Results of the three‐way ANOVA test.

Source	SS	*df*	Mean square	*F*	*p* value
Bonding substrate (enamel or dentin)	5.217	1	5.217	2.937	0.091
Mode of application of adhesive bonding (SE or E&R)	0.004	1	0.004	0.002	0.963
Type of composite resin (conventional or bulk‐fill)	127.588	1	127.588	71.813	< 0.001*
Bonding substrate × Mode of application of adhesive bonding	768.118	1	768.118	432.335	< 0.001*
Bonding substrate × Type of composite resin	3.180	1	3.180	1.790	0.185
Type of composite resin × Mode of application of adhesive bonding	3.921	1	3.921	2.207	0.142
Type of composite resin × Mode of application of adhesive bonding × Bonding substrate	0.766	1	0.766	0.431	0.513
Error	127.920	72	1.777	—	—
Total	32,546.209	80	—	—	—

Abbreviations: ANOVA, analysis of variance; SS, the sum of squares; *df*, degrees of freedom; *F*, *F* statistic.

* Significant at *p* < 0.05.

The group that demonstrated the highest μSBS values for enamel was the one utilizing the E&R mode with the bulk‐fill composite resin (group 3). Conversely, for dentin, the highest μSBS values were observed in the SE mode with the bulk‐fill composite resin (group 8). According to the *t* tests, when bonding either the bulk‐fill or conventional composite resin to enamel, the μSBS of the E&R group was significantly higher than that of the SE group (*p* < 0.001). However, when bonding to dentin, the μSBS of the SE group was significantly higher than that of the E&R group (*p* < 0.001).

Additionally, the μSBS of the bulk‐fill composite resin when bonded to enamel or dentin exhibited a significantly higher value compared to that of the conventional composite resin, regardless of whether the E&R or SE mode of application was employed (*p* < 0.001). When comparing the mode of application of adhesive bonding to enamel or dentin, the E&R mode displayed significantly higher bond strength to enamel than the SE mode. Conversely, the SE mode resulted in significantly higher bond strength to dentin compared to the E&R mode, regardless of the type of composite resin used (conventional or bulk‐fill) (*p* < 0.001).

**Figure 5:**
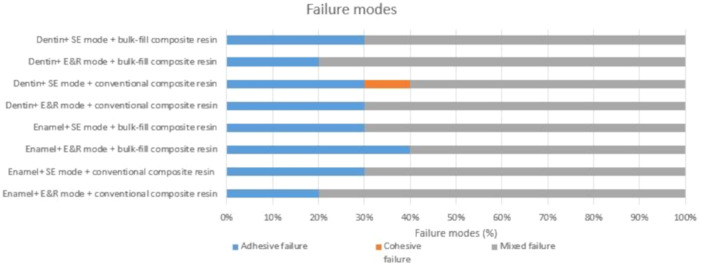
Distribution of failure mode frequency.

**Figure 6:**
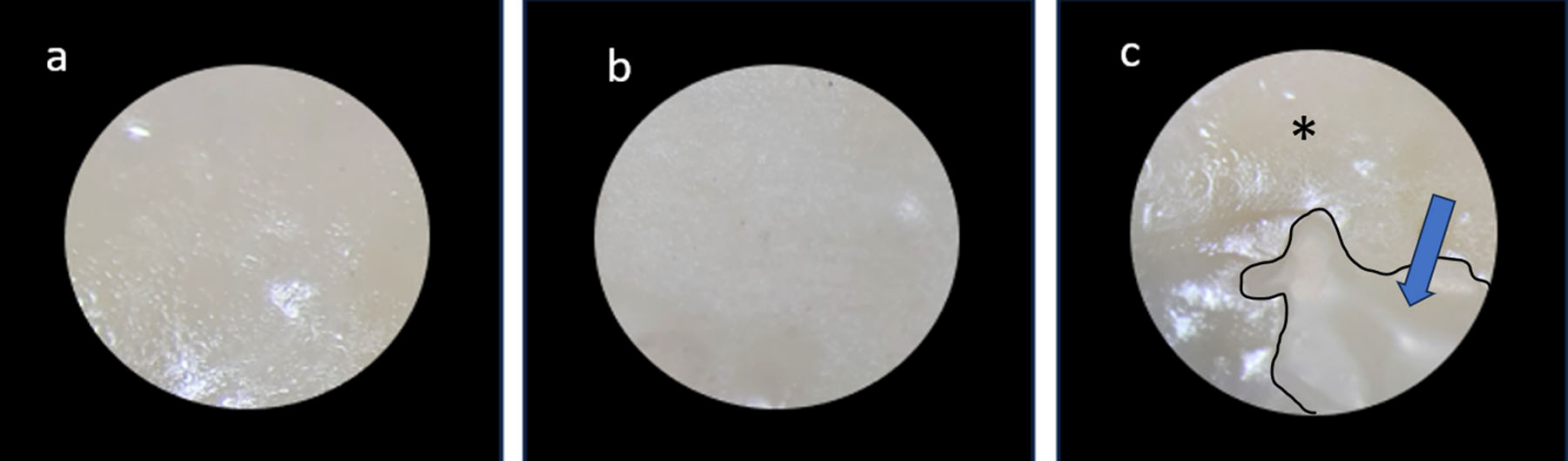
Instances of various fracture modes observed through a stereomicroscope (×40) during the microshear bond strength test conducted in the present study. (a) Adhesive failure; (b) cohesive failure in composite resin; and (c) mixed failure. *The adhesive and arrows point to the composite resin.

Figure 5 presents the results regarding failure modes. Mixed failure was the predominant mode observed in all study groups. Cohesive failure within the composite resin was observed in only one instance, specifically in group 6. Figure 6 displays the representative examples of different fracture modes that were observed during the μSBS test in the current study.

## 4 Discussion

This study aimed to evaluate the impact of different application modes of universal adhesives on the bond strength of bulk‐fill composite resins in primary teeth. The null hypothesis proposed that there would be no significant differences in μSBS when employing different application modes of the universal adhesive system to bond bulk‐fill composite resin to enamel and dentin surfaces of primary teeth. However, based on the study's findings, the null hypothesis was rejected. The bond strength of bulk‐fill composite resin was significantly higher than that of conventional composite resin. Moreover, when bonding composite resin to primary enamel, the E&R mode resulted in significantly higher bond strength compared to the SE mode. Conversely, the SE mode yielded significantly higher bond strength compared to the E&R mode when bonding composite resin to dentin.

The μSBS test is a reliable and straightforward method for assessing bond strength. It offers the advantage of allowing multiple specimens to be prepared from the same tooth due to the small size of the tested areas. Unlike macroshear tests, which have limitations such as uneven stress distribution in the loaded cross‐section and mixed loading mode, the μSBS test does not suffer from these drawbacks. In macroshear tests, when the loads are significantly lower than the shear strength, cohesive failure in the dentin substrate tends to occur instead of failure at the interface between the restorative material and dentin (Sadat‐Shojai et al. 2010). Therefore, in this study, the μSBS test was utilized to compare the effectiveness of a bulk‐fill composite resin and a conventional composite resin on primary teeth.

The demand for simplified and efficient restorative techniques has grown, particularly in the context of primary teeth. These techniques involve the placement of composite resin in bulk or the utilization of single‐step self‐etching adhesives (Ilie et al. 2014). With this in mind, the objective of the present study was to compare the bonding effectiveness of a bulk‐fill composite resin and a conventional composite resin when combined with different application modes of a universal adhesive system in primary teeth.

Conventional composite resins have been extensively used for restorative procedures in primary teeth. However, the conventional 2 mm‐incremental technique employed with these resins presents several challenges. These challenges include prolonged application time, the potential for voids or contamination between layers, and the risk of bonding failure between layers (Kim et al. 2015). To overcome these issues, the use of bulk‐fill composite resins has gained popularity among dentists in recent years (Oter, Deniz, and Cehreli 2018). Bulk‐fill composite resins are formulated with larger filler particles (size > 20 μm) and lower filler content compared to conventional composite resins. This composition enhances their translucency and allows for a greater depth of cure, reaching up to 4 mm (Ilie, Bucuta, and Draenert 2013). Moreover, bulk‐fill composite resins have demonstrated various advantages, such as reduced cusp deflection, decreased polymerization shrinkage stress, and favorable bond strengths and marginal quality regardless of the filling technique or cavity configuration (Moorthy et al. 2012; Van Ende et al. 2013).

In recent years, many studies have been carried out to investigate the effectiveness of using bulk‐fill composite resins for restorations in primary teeth (Oter, Deniz, and Cehreli 2018; Eltoum et al. 2019; Ghajari et al. 2020). The main benefit of employing bulk‐fill composite resins in primary teeth is the reduction in clinical time, which is especially valuable in the field of pediatric dentistry (Gindri et al. 2022). Previous research has demonstrated that the marginal integrity of bulk‐fill composite resin restorations in primary molars is not statistically different when compared to incrementally placed conventional composite resins (Eltoum et al. 2019). Additionally, bulk‐fill composite resin has been shown to significantly enhance the fracture resistance of pulpotomized primary molars compared to conventional composite resin (Ghajari et al. 2020). Moreover, a randomized clinical trial conducted previously reported similar successful performance at a 1‐year follow‐up for both conventional and bulk‐fill composite restorations in class 1 cases (Oter, Deniz, and Cehreli 2018). Another recently published randomized clinical trial found comparable clinical performance with a 30% reduction in required clinical time for class II lesion restorations in primary molars (Gindri et al. 2022).

In this particular research, x‐tra fil (Vocco) was used as the bulk‐fill composite resin. Previous investigations have indicated that x‐tra fil has the highest hardness ratios among the bulk‐fill composites tested, ensuring adequate conversion after polymerization in 4 mm layers (Colombo et al. 2020). Moreover, x‐tra fil exhibited the least reduction in microhardness value when exposed to acid storage (Colombo et al. 2020). The present study's findings reveal that when applied to enamel and dentin in primary teeth, the bulk‐fill composite resin displayed superior bond strength compared to conventional composite resin. This result aligns with a previous study that reported similar or improved performance of bulk‐fill materials compared to nanohybrid composite resins for restorations in primary and permanent teeth after 1 year (Ilie et al. 2014). The higher bond strength observed in the bulk‐fill composite resin may be attributed to a potential reason, which is the lower polymerization shrinkage stress when compared to conventional composite resins (Cidreira Boaro et al. 2019). The decreased polymerization shrinkage stress of bulk‐fill composite resin helps alleviate adverse effects on the bonding interface, thereby leading to an increase in bond strength (Cidreira Boaro et al. 2019). Furthermore, this outcome could be ascribed to the enhanced ability of the bulk‐fill material to wet and adapt to the tooth substrate in comparison to conventional composite resin. This aspect warrants further investigation in future research (Ilie et al. 2014).

The objective of this study was to examine how the application mode of a universal adhesive system affects the bond strength of bulk‐fill and conventional composite resins in primary teeth. Universal adhesives are provided in a single bottle, enabling their utilization in both E&R and SE modes (Memarpour et al. 2018). However, previous research has yielded conflicting findings regarding the impact of the application mode of universal adhesives (Lenzi et al. 2013; Nicoloso 2016; Thanaratikul, Santiwong, and Harnirattisai 2016; Kim et al. 2017). One study, for instance, reported higher bond strength in primary tooth dentin when utilizing a universal adhesive bonding system (in either E&R or SE mode) compared to a two‐step E&R adhesive bonding agent (Memarpour et al. 2018). Thanaratikul et al. discovered no disparity in bond strength when comparing the two application modes, E&R and SE, of Single Bond Universal Adhesive while bonding to primary dentin (Thanaratikul, Santiwong, and Harnirattisai 2016). In a systematic review, it was concluded that the etching technique did not impact the bond strength of mild universal adhesives to primary dentin, even after undergoing the aging process (Fröhlich et al. 2021). However, Ghajari et al. reported improved outcomes for the SE strategy (Ghajari et al. 2019).

In this particular investigation, the universal adhesive bonding system utilized was Gluma Bond Universal. This adhesive comprises two acidic polymerizable monomers: 10‐methacryloxydecyl dihydrogen phosphate (10 MDP) and 4‐methacryloxyethyl trimellitic acid (4‐MET). Both 4‐MET and 10‐MDP operate through similar bonding mechanisms with dentin and can chemically interact with the residual hydroxyapatite present after the adhesive bonding process (Sanabe et al. 2009). This interaction results in the formation of 10‐MDP‐ or 4‐MET‐Ca salts, which then deposit on dental hard tissue, forming hydrophobic self‐assembled nanolayers (Sanabe et al. 2009). As a result of this reaction, the formation of 10‐MDP‐ or 4‐MET‐Ca salts occurs, which subsequently deposit onto the dental hard tissue, leading to the creation of hydrophobic self‐assembled nanolayers (Sanabe et al. 2009). Typically, a hydrophobic adhesive layer demonstrates superior mechanical properties and is more resilient against degradation factors when compared to a hydrophilic layer (Takamizawa et al. 2015). Consequently, Gluma Bond Universal can establish a hybrid layer by combining both chemical bonding and micromechanical locking mechanisms (Sanabe et al. 2009).

Based on the findings of this study, the utilization of the universal adhesive system in the SE mode demonstrated superior dentin bond strength compared to the E&R mode. This aligns with the results of a prior investigation (Ghajari et al. 2019). The reason for this result can be explained by the fact that when phosphoric acid is applied before the adhesive in the E&R mode, it causes a more significant demineralization of dentin (3–6 μm). This depth of demineralization surpasses the anticipated penetration capacity of functional monomers into the demineralized dentin (Isolan 2014). Moreover, Gluma Bond Universal exhibits a pH of 1.8, placing it in the category of SE adhesives with an intermediate level of strength (Van Meerbeek et al. 2011). As a result, the application of phosphoric acid before the acidic adhesive can cause excessive etching, resulting in the removal of remaining hydroxyapatite from the collagen network and a decrease in the potential for chemical adhesion (Ahn et al. 2015). Alternatively, there is a possibility that the adhesive monomers may not effectively penetrate the exposed collagen fibers and dentinal tubules, leading to the formation of shorter resin tags (Isolan 2014). Hence, when a universal adhesive with a pH lower than 2 is utilized in the E&R mode on dentin, there is a potential for reduced micromechanical interlocking to take place (Ahn et al. 2015). Moreover, the residual exposed collagen network following the etching process possesses a low surface energy, which can result in a decrease in the strength of the dentin bond (Ghajari et al. 2019).

Primary dentin exhibits distinct microstructural features, including larger diameters in both peritubular and intertubular dentin, lower concentrations of phosphate and calcium in these regions, higher tubular density, and increased reactivity in comparison to permanent dentin (Lenzi et al. 2013). Therefore, when universal adhesives are utilized in the E&R mode on primary dentin, a more significant impact of the adhesive systems is witnessed in comparison to permanent teeth (Ghajari et al. 2019). This effect results in deeper and more severe demineralization of the intertubular dentin in primary teeth compared to permanent teeth (Ghajari et al. 2019).

Unlike the results obtained in the current study, which demonstrate the superior performance of the universal adhesive system when applied in the SE mode on primary dentin compared to the E&R mode, Thanaratikul et al. found no discernible difference in dentin bond strength between these two application modes of universal adhesive (Thanaratikul, Santiwong, and Harnirattisai 2016). The contrasting outcomes might be attributed to differences in the pH and composition of the universal adhesives employed. Thanaratikul et al. utilized Single Bond Universal Adhesive, which falls under the category of mild universal adhesives (Thanaratikul, Santiwong, and Harnirattisai 2016). Nonetheless, in the current investigation, Gluma Bond Universal, an intermediate‐strength SE adhesive, was utilized on primary dentin. Previous studies have suggested that the pH of universal adhesives can impact their performance (Cuevas‐Suarez 2019). Thus, the bond strength of universal adhesives in primary teeth may be influenced by the varying compositions and pH levels of different adhesives. This highlights the need for future studies to assess additional universal adhesives with different pH values. Additionally, discrepancies in study results may arise due to differences in tooth types, adhesive application methods (such as SE or E&R modes), and evaluation techniques employed.

The results of the current study demonstrated that the E&R mode yielded significantly higher bond strength between the universal adhesive and enamel compared to the SE mode. This observation aligns with a previous study that also showed enhanced bond strength values with universal adhesives after phosphoric acid etching of enamel in permanent teeth, regardless of the adhesive's pH (Cuevas‐Suarez 2019). Similarly, Antoniazzi and colleagues discovered that the selective application of acid etching on enamel in primary teeth resulted in enhanced bond strength of a universal adhesive system. This finding corresponds with the outcomes of the current study (Antoniazzi et al. 2016). The enhancement in bond strength can be attributed to the formation of larger and smaller pores on the enamel surface as a result of applying phosphoric acid. This process improves the surface's ability to be wetted by the adhesive and increases the bonding area on the enamel surface (Thanaratikul, Santiwong, and Harnirattisai 2016).

Moreover, the present study revealed that the E&R technique yielded lower bond strength to primary dentin in comparison to enamel. The diminished bond strength of the universal adhesive on dentin, in the E&R mode compared to enamel, could be attributed to the lower mineral content of dentin when compared to enamel (Angker et al. 2004). Pretreating dentin with acid etching before the application of an acidic universal adhesive system can result in more extensive demineralization of dentin and incomplete penetration, akin to E&R adhesive systems. This primarily contributes to a reduction in dentin bond strength (Angker et al. 2004; Betancourt, Baldion, and Castellanos 2019).

In terms of the distribution of bond strength values, there was a relatively equal distribution among the different groups in the study. However, the prevalence of mixed failure, followed by adhesive failure, was consistently observed across all groups. This finding is consistent with previous studies that have also identified mixed and adhesive failure modes as the primary types of failure in primary teeth (Bolaños‐Carmona et al. 2006; Mosharrafian and Sharifi 2016). The occurrence of mixed and adhesive failure modes can be attributed to multiple factors, including the characteristics of the substrates (enamel and dentin) and the specific adhesive system employed. The mixed failure mode indicates a combination of cohesive and adhesive failure, suggesting that the bond between the composite resin and the tooth structure experienced both internal and interfacial stress. On the other hand, the adhesive failure mode indicates that the bond primarily failed at the interface between the adhesive and the tooth substrate. The prevalence of mixed and adhesive failure modes in primary teeth is likely influenced by the unique properties of primary tooth structure. These properties include variations in enamel thickness, dentin composition, and the presence of a more porous enamel surface. Such factors can impact the bonding mechanism and contribute to the observed failure modes.

The reduction of chair time in pediatric dentistry has always been a crucial aspect (Oter, Deniz, and Cehreli 2018). Additionally, there has been an increasing demand for esthetic and long‐lasting restorations (Oter, Deniz, and Cehreli 2018). According to the results obtained from this study, bulk‐fill composite resin exhibited superior bond strength when compared to conventional composite resin. Consequently, when dealing with uncooperative children, the use of bulk‐fill composite resins can be a favorable choice for esthetic restorations in primary teeth. Regarding the etching technique, pre‐etching the enamel before the application of universal adhesive improved the bond strength to primary teeth enamel in this study. Therefore, it is recommended to selectively acid etch the enamel when using universal adhesives on primary teeth.

Nevertheless, it is crucial to acknowledge certain limitations of this study. First, only a single commercial universal adhesive was examined, and it is advisable for future studies to assess multiple commercial universal adhesive systems with varying pH values. Furthermore, further laboratory experiments and randomized clinical trials with extended follow‐up periods are necessary to validate the findings obtained in this study.

## 5 Conclusion

The findings of this study indicate that bulk‐fill composite resin exhibited superior performance compared to conventional composite resin in primary teeth, making it a viable choice for expedited restorations. In terms of the etching strategy, the SE mode of the universal adhesive demonstrated better results than the E&R mode on primary dentin. However, prior enamel etching enhanced the bond strength of the universal adhesive to primary enamel, underscoring the recommendation for selective acid etching of primary enamel.

## Author Contributions

Zahra Jowkar and Ali Nozari conceived the ideas; all authors collected the data; all authors analyzed the data; and Zahra Jowkar and Ali Nozari led the writing.

## Conflicts of Interest

The authors declare no conflicts of interest.

## Data Availability

The data that support the findings of this study are available on request from the corresponding author. The data are not publicly available due to privacy or ethical restrictions.
